# Cooperative lipolytic control of neuronal triacylglycerol by spastic paraplegia-associated enzyme DDHD2 and ATGL

**DOI:** 10.1016/j.jlr.2023.100457

**Published:** 2023-10-11

**Authors:** Peter Hofer, Gernot F. Grabner, Mario König, Hao Xie, Dominik Bulfon, Anton E. Ludwig, Heimo Wolinski, Robert Zimmermann, Rudolf Zechner, Christoph Heier

**Affiliations:** 1 Institute of Molecular Biosciences, University of Graz, Graz, Austria; 2 State Key Laboratory of Natural Medicines, Key Laboratory of Drug Metabolism and Pharmacokinetics, China Pharmaceutical University, Nanjing, China; 3 BioHealth Field of Excellence, University of Graz, Graz, Austria; 4 BioTechMed-Graz, Graz, Austria

**Keywords:** Lipolysis and fatty acid metabolism, brain lipids, enzymology, lipase, triacylglycerol, lipid droplets, neurons, spastic paraplegia

## Abstract

Intracellular lipolysis—the enzymatic breakdown of lipid droplet-associated triacylglycerol (TAG)—depends on the cooperative action of several hydrolytic enzymes and regulatory proteins, together designated as lipolysome. Adipose triglyceride lipase (ATGL) acts as a major cellular TAG hydrolase and core effector of the lipolysome in many peripheral tissues. Neurons initiate lipolysis independently of ATGL via DDHD domain-containing 2 (DDHD2), a multifunctional lipid hydrolase whose dysfunction causes neuronal TAG deposition and hereditary spastic paraplegia. Whether and how DDHD2 cooperates with other lipolytic enzymes is currently unknown. In this study, we further investigated the enzymatic properties and functions of DDHD2 in neuroblastoma cells and primary neurons. We found that DDHD2 hydrolyzes multiple acylglycerols in vitro and substantially contributes to neutral lipid hydrolase activities of neuroblastoma cells and brain tissue. Substrate promiscuity of DDHD2 allowed its engagement at different steps of the lipolytic cascade: In neuroblastoma cells, DDHD2 functioned exclusively downstream of ATGL in the hydrolysis of *sn*-1,3-diacylglycerol (DAG) isomers but was dispensable for TAG hydrolysis and lipid droplet homeostasis. In primary cortical neurons, DDHD2 exhibited lipolytic control over both, DAG and TAG, and complemented ATGL-dependent TAG hydrolysis. We conclude that neuronal cells use noncanonical configurations of the lipolysome and engage DDHD2 as dual TAG/DAG hydrolase in cooperation with ATGL.

Triacylglycerol (TAG) storage in cytosolic lipid droplets (LDs) constitutes a major cellular reservoir of energy, signaling lipids, and structural components. The utilization of these stores requires TAG breakdown via the concerted action of hydrolytic enzymes, a process commonly referred to as intracellular lipolysis. According to prevalent models, lipolysis occurs in a step-wise manner by the consecutive hydrolysis of ester bonds, each step being catalyzed by a different enzyme (1, 2). Mutual enzymatic substitutions are partially possible due to overlapping substrate specificities. The enzymology of lipolysis has been initially established in adipocytes, which constitute the major TAG reservoir of the body. Hormonal stimulation of adipocyte lipolysis induces the complete breakdown of TAGs into FAs and glycerol. In adipocytes, these components are then released into the bloodstream for transport into other tissues. Efficient TAG breakdown in adipocytes requires three enzymes: adipose triglyceride lipase (ATGL) initiates intracellular lipolysis via the selective hydrolysis of TAG into FAs and diacylglycerol (DAG) (3, 4, 5). Subsequently, DAG is hydrolyzed to monoacylglycerol (MAG) by hormone-sensitive lipase (HSL) (6, 7) and MAG is converted to glycerol by monoglyceride lipase (MGL) (8). Together with a dynamic set of accessory proteins modulating enzyme activity, stability, and localization, the three lipases ATGL, HSL, and MGL constitute the so-called “lipolysome” (9). The concerted action of the lipolysome ensures adequate FA supply matching the current metabolic state.

The characterization of mutant animal models revealed that ATGL initiates lipolysis in multiple nonadipose cell types including enterocytes, hepatocytes, immune cells, and myocytes (5, 10, 11, 12, 13, 14, 15, 16). However, the precise configuration of the “lipolysome” in these cells differs from adipocytes and is often incompletely understood. Knockout of HSL elevates DAG in adipocytes and myocytes but not hepatocytes (6) where it is weakly expressed (17). Accordingly, several other enzymes have been implicated in the hydrolysis of TAG, DAG, and MAG, in particular in nonadipose tissues (18, 19, 20, 21, 22, 23). Thus, it appears that, due to the particular tissue distribution patterns of lipases and regulatory proteins, the configuration of the “lipolysome” is variable and tissue-specific.

A recently identified lipolysome member, DDHD-domain containing 2 (DDHD2, also annotated as KIAA0725p or intracellular phospholipase A_1_ (iPLA_1_γ)), acts as a major neuronal TAG lipase and represents a critical enzyme for lipid homeostasis in the central nervous system (24, 25). DDHD2 shows ubiquitous tissue expression with highest levels in brain, testis, and skeletal muscle (26). Together with its two paralogs Sec23 interacting protein (Sec23ip, also annotated as p125 or iPLA_1_β) and DDHD1 (also annotated as PA-PLA_1_ or iPLA_1_α), DDHD2 constitutes the iPLA_1_ protein family (27). A common structural feature of all three iPLA_1_ family members is their ∼180 amino acid-spanning DDHD domain characterized by four conserved residues (three Asp and one His residue giving rise to the name). In addition to its TAG hydrolase function, DDHD2 has been assigned roles in phospholipid breakdown (26, 28), vesicle trafficking (29, 30), and mitochondrial function (31, 32).

Patients with loss-of-function mutations in the human *DDHD2* gene suffer from a complex hereditary spastic paraplegia (SPG54) that includes lower limb weakness, gait impairment, and intellectual disability (33, 34, 35, 36). A characteristic of those patients is the accumulation of neutral lipid in certain brain regions, which can be visualized by NMR and serve as diagnostic marker to discriminate against other SPGs. Knockout or pharmacological inhibition of DDHD2 in mice recapitulates this phenomenon as it elevates brain TAG levels and induces LD formation in neurons (24). This phenotype differs from ATGL knockout mice, which accumulate LDs in cerebrovascular cells but do not exhibit extensive lipid accumulation in neurons (37). This suggests that the enzymology of neuronal lipolysis differs fundamentally from other tissues. Whether and how DDHD2 cooperates with other hydrolytic enzymes in lipolysis is currently unknown.

We compared the enzymological properties of the murine DDHD family with ATGL and HSL and found that DDHD2 has a unique capacity to hydrolyze acylglycerols in vitro with a similar potency as ATGL and HSL. Using neuroblastoma cells and primary neurons as model systems, we found that DDHD2 engages in the lipolysome as dual DAG/TAG lipase and cooperates with ATGL in neuronal lipolysis.

## Materials and Methods

### Animals

Animal experiments were approved by the Austrian Federal Ministry for education, science, and research (protocol number BMBWF-66.007/0008-V/3 b/2018) and the ethics committee of the University of Graz and were conducted in compliance with the council of Europe Convention (ETS 123). Animal experiments were designed and performed in accordance with the principles of the three Rs and the ARRIVE guidelines and conform to national and EU directives about animal rights. C57BL/6J mice were bred and maintained on a regular dark light cycle (14 h light, 10 h dark) at 22 ± 1°C in a barrier facility in specific pathogen-free quality. A standard laboratory chow diet (R/M-H Extrudate, V1126-037, Ssniff Spezialdiäten GmbH, Soest, Germany) and drinking water were provided ad libitum. Tissues were sampled from male mice aged 3–6 months.

### Molecular cloning

The coding sequences of murine *Ddhd2*, *Ddhd1*, and *Sec23ip* were inserted into pcDNA4/HisMax C (Thermo Fisher Scientific, Waltham) thereby equipping the respective proteins with a N-terminal His_6_-tag. To this end, the coding sequences were amplified by PCR from C57BL/6J testis cDNA using the primers 5′-GATCGGATCCATGTCATCGGGGGAATCAC-3′ and 5′-GATCCTCGAGTTACTGTAAAGGCTGATCAAG-3’ (for *Ddhd2*), 5′-GATCGAATTCATGAACTACCCGGGCCGCGG-3′ and 5′-CATGCTCGAGTCAGAGTGAACCTAGGCTGG-3’ (for *Ddhd1*), 5′GATCGGTACCAATGGCGGATAGGAAGGCTAAC-3′ and 5′GATCCTCGAGTCAATGCTGGGGCTGCTCTGG-3’ (for *Sec23ip*). PCR was performed using the Phusion™ high-fidelity DNA polymerase (ThermoFisher Scientific, Waltham) with 0.33 μM primers and 30 μM dNTPs in HF buffer and the following amplification parameters: initial denaturation for 30 s at 98°C; 35 cycles of denaturation for 20 s at 98°C, annealing for 30 s at 60°C, extension for 90 s at 72°C; final extension for 5 min at 72°C. The coding sequence of human *DDHD2* was amplified from HepG2 cDNA using the primers 5′GATCGGATCCATGTCATCAGTGCAGTCAC-3′ and 5′-GCTACTCGAGTTACTGTAAAGGCTGATCAA-3′ and the same parameters as above. PCR products and pcDNA4/HisMax C vector DNA (Thermo Fisher Scientific, Waltham) were digested with BamHI and XhoI (for murine *Ddhd2* and human *DDHD2*), EcoRI and XhoI (for *Ddhd1*), KpnI and XhoI (for *Sec23ip*), and ligated using the T4 DNA Ligase (NEB, Ipswich) according to the manufacturer’s instructions. Constructs for the expression of murine His_6_-ATGL and murine His_6_-Hsl have been previously described (38). The coding sequences of murine *Atgl* and *Ddhd2* were inserted into pECFP-N1 (Takara, Kyoto, Japan) equipping the respective proteins with a C-terminal ECFP tag. To this end, the coding sequences of murine *Atgl* and *Ddhd2* were amplified using the primers 5′-GATCCTCGAGGCCACCATGTTCCCGAGGGAGACCAA-3′ and 5′-GACTCCGCGGGCAAGGCGGGAGGCCAGGT-3’ (for *Atgl*) and 5′-ATTACTCGAGGCCACCATGTCATCGGGGGAATCACA-3′ and 5′-GCTACCGCGGCTGTAAAGGCTGATCAAGGA-3’ (for *Ddhd2*) and the parameters described above. PCR products and vector DNA were digested with XhoI and SacII and ligated using the T4 DNA ligase (NEB, Ipswich) according to the manufacturer’s instructions. A vector expressing shRNA targeting Ddhd2 was generated by inserting an oligonucleotide into the lentiviral vector pLKO.1 Puro (Addgene plasmid # 8453). To this end, the oligonucleotides 5′-CCGGATGTTTGGTTCGCTTATATCTCGAGATATAAGCGAACCTAAACTATTTTTTG-3′ and 5′- AATTCAAAAAATGTTTGGTTCGCTTATATCTCGAGATATAAGCGAACCTAAACTAT-3′ were incubated for 5 min at 95°C in NEB buffer 2 followed by 10 min at 70°C. Afterward, the reaction was slowly cooled to room temperature. The vector was digested with AgeI and EcoRI, and the annealed oligonucleotides were ligated using the T4 DNA ligase (NEB, Ipswich) according to the manufacturer’s instructions. A pLKO.1 Puro vector expressing scramble shRNA was a gift from David Sabatini (Addgene plasmid #1864) (39). All constructs were transformed into NEB 5-alpha Competent *Escherichia coli* cells (NEB, Ipswich) and isolated using the NucleoBond™ Xtra Midi Kit (Macherey-Nagel, Düren, Germany).

### Cell culture

COS-7 cells (CRL-1651, ATCC) were maintained in low glucose DMEM (Thermo Fisher Scientific, #11885084) supplemented with 10% fetal calf serum (Biowest, Nuaillé, France), 50 U/ml penicillin, and 50 μg/ml streptomycin (Thermo Fisher Scientific, #15070063) at standard conditions (37°C, 95% humidified air, 7% CO_2_). COS-7 cells were transfected using Metafectene (Biontex, Munich, Germany) according to the manufacturer’s instructions. Neuro-2a cells (CLL-131, ATCC) were maintained in MEM (Thermo Fisher Scientific, #31095029) supplemented with 10% fetal calf serum (Biowest), 50 U/ml penicillin, 50 μg/ml streptomycin (Thermo Fisher Scientific, #15070063), 1 mM sodium pyruvate, and 1% non-essential amino acids (Thermo Fisher Scientific, #11140050). Neuro-2a cells were transfected using Lipofectamine 3000 (Thermo Fisher Scientific, #L3000015) according to the manufacturer’s instructions.

Primary neurons were isolated as previously described (40). In brief, brains from 15.5-day-old embryos were dissected in papain solution (Merk, #P4762) after meninges were removed. Neurons were seeded in cultivation dishes coated with 50 μg/ml poly-D-lysine and cultivated in Neurobasal medium (Thermo Fisher Scientific, #21103049) containing 2% B-27 supplement (Thermo Fisher Scientific, #17504044), 100 IU/ml penicillin/streptomycin (Thermo Fisher Scientific, #15070063), 100 μg/ml primocin (Invivogen, #ant-pm-2), and 0.5 mM glutamine (Thermo Fisher Scientific, # 25030024). Neurons were maintained at standard conditions (37°C, 95% humidified air, 7% CO2), half of the nutrition medium was exchanged every 2 days, and experiments were performed 7 days after neuron dissection.

### Gene silencing

Viral particles were produced using the Lenti-X™ Lentiviral Expression System (Takara, Kyoto, Japan). In brief, HEK-293T cells (CRL-3216, ATCC) were cotransfected with pLKO.1 Puro containing shRNA targeting Ddhd2 or scramble shRNA and the Lenti-X™ HTX packaging mix. After 48 h, the lentiviral supernatant was used to transduce Neuro-2a cells by spinoculation at 1,200 *g* and 32°C for 1 h in presence of 8 μg/ml polybrene. Another 24 h later, transduced cells were selected by adding puromycin at a concentration of 2.5 μg/ml.

### Cell lysis and protein determination

Cells were cultivated on 100 mm^2^ dishes and harvested by trypsinization or scraping according to the experimental requirements. Cells were collected by brief centrifugation and washed with PBS (137 mM NaCl, 2.7 mM KCl, 10 mM Na_2_HPO_4_, 1.8 mM KH_2_PO_4_). Afterward, cells were resuspended in solution A (250 mM sucrose, 1 mM EDTA, 1 mM dithiothreitol, pH 7.0) supplemented with 20 μg/ml leupeptin, 2 μg/ml antipain, and 1 μg/ml pepstatin and disrupted by sonication. Perinuclear supernatants were prepared by centrifugation for 10 min at 4°C and 1,000 *g*. Protein concentration was measured with the Bradford protein assay (Bio-Rad Laboratories, Hercules) using bovine serum albumin (BSA) as standard.

### Western blotting

Protein samples were boiled in 6× sample buffer (0.3 M Tris HCl pH 6.8%, 15% 2-mercaptoethanol, 12% SDS, 60% glycerol, bromophenol blue) and resolved in 10% acrylamide gels at 15 mA. Proteins were blotted onto polyvinylidene difluoride membranes (Carl Roth, Karlsruhe, Germany) at 200 mA in 10 mM CAPS pH 11.0 supplemented with 10% methanol. The membranes were blocked for one hour with 10% blotting-grade milk powder (Carl Roth) dissolved in TST (50 mM Tris HCl pH 7.4, 150 mM NaCl, and 1% Tween-20) prior to incubation with the primary antibody solution overnight at 4°C. Bound antibodies were detected with horseradish peroxidase anti-mouse IgG conjugate (Cytiva, Marlborough), anti-rabbit IgG conjugate (#PI-1000; Vector laboratories, Burlingame), or anti-guinea pig IgG conjugate (#6090-04, Southern Biotechnology, Birmingham) and visualized by enhanced chemiluminescence detection (Clarity Western ECL Blotting Substrate, Bio-Rad Laboratories, Hercules) in a ChemiDoc Touch Imaging system (Bio-Rad Laboratories, Hercules). The following primary antibodies were used: Guinea pig anti-PLIN2 (#GP40; Progen, Frankfurt, Germany), rabbit anti-DDHD2 (#25203-1-AP, Proteintech, Manchester, UK), rabbit anti-GAPDH (#2118), rabbit anti-IRE1α (#3294), rabbit anti-ATGL (#2138), rabbit anti-HSL (#4107, all Cell Signaling Technology), and mouse anti-His_6_ (#27-4710-01, GE Healthcare, Buckinghamshire, UK).

### In vitro TAG hydrolase activity assay

TAG hydrolase activities of cell lysates were determined as described in (41).

### In vitro DAG hydrolase activity assay

DAG hydrolase activities of cell lysates were determined analogous to TAG hydrolase activities with the following modifications. The substrate contained 2 μCi/ml 1,3-dioleoyl-[9,10-^3^H]-*rac*-glycerol (American Radiolabeled Chemicals, St. Louis), 0.3 mM 1,3-dioleoyl-*rac*-glycerol, and 45 μM phosphatidylcholine (PC)/phosphatidylinositol (3:1 M ratio). FAs were extracted and quantified as above. For the determination of the enzymatic activities of overexpressed enzymes, the radioactive tracer was omitted and the release of FA was quantified colorimetrically using the NEFA-HR(2) Assay Kit (Fujifilm Wako Chemicals, Neuss, Germany) according to the manufacturer’s instructions.

### In vitro MAG hydrolase activity assay

The MAG substrate was prepared by sonicating a mixture of 4.09 mM 1-oleoyl-*rac*-glycerol, 6.4 mM CHAPS, 1 mM EDTA, and 100 mM bis-tris propane using a VirTis Virsonic 475 Ultrasound Cell Disruptor (20 s, 10% output power). Fifty microliters of the MG substrate were incubated with 50 μl of cell lysates at 37°C for 30 min. FAs were quantified colorimetrically using the NEFA-HR(2) Assay Kit (Fujifilm Wako Chemicals, Neuss, Germany) according to the manufacturer’s instructions.

### In vitro PC hydrolase activity assay

1,2-dioleoyl-*sn*-glycero-3-phosphocholine was emulsified at a final concentration of 0.3 mM by sonication as described for the TAG hydrolase assay. Cellular protein was incubated with the substrate for 60 min at 37°C and the release of FA was quantified colorimetrically using the NEFA-HR(2) Assay Kit (Fujifilm Wako Chemicals, Neuss, Germany) according to the manufacturer’s instructions.

### ^14^C-oleic acid incorporation experiments

Neuro-2a cells were seeded in 6-well plates at 3 × 10^5^ cells/well. Twenty-four hours later, cells received fresh cultivation medium supplemented with 400 μM oleic acid complexed to 135 μM FA-free BSA, 0.2 μCi/well [1-^14^C] oleic acid (American Radiolabeled Chemicals, St. Louis), and small molecule inhibitors or DMSO as vehicle control.

Sixteen hours later, cells were washed twice with PBS and lipids were extracted with 1 ml hexane/isopropanol (3/2, v/v) on a rocking shaker for 10 min. Lipid extracts were dried under a stream of N_2_ gas, dissolved in CHCl_3_/methanol (2/1, v/v), and spotted onto silica gel-coated aluminum TLC sheets (Merck, Darmstadt, Germany). The sheets were developed in CHCl_3_/acetone/acetic acid (90/8/1, v/v/v), sprayed with scintillation cocktail (ROTISZINT eco plus, Carl Roth)/methanol/H_2_O (4/1/1, v/v/v), and exposed to light-sensitive films (Amersham Hyperfilm ECL, Cytiva) for 18 h at −80°C. Bands corresponding to TAG and DAG were excised from the TLC sheets, immersed in scintillation cocktail for 24 h, and radioactivity was determined by liquid scintillation counting in a Tri-Carb 2900TR liquid scintillation analyzer (PerkinElmer, Waltham). The dried and delipidated cells were lysed in NaOH/SDS (0.3 N/0.1%) overnight at room temperature. Protein concentration of the lysates was measured with the Pierce™ BCA Protein Assay Kit (Thermo Fisher Scientific) according to the manufacturer’s instructions. BSA was used as standard. TAG and DAG levels were calculated relative to total cellular protein content. To analyze FA release, oleic acid-loaded Neuro-2a cells received fresh cultivation medium supplemented with 2% FA-free BSA as FA scavenger. Four hours later, radioactivity of cell supernatants was determined by liquid scintillation counting. Intracellular TAG levels were determined as above.

### Small molecule inhibitors

The ATGL inhibitor Atglistatin was synthesized as described and used at 40 μM (42). The HSL inhibitor 76-0079 was obtained from Novo Nordisk (Bagsværd, Denmark, Cat.No. #NNC 0076-0000-0079) and used at 1 μM. The DDHD2 inhibitor KLH45 was obtained from Sigma-Aldrich (St. Louis#SML1998) and used at 100 nM.

### Subcellular fractionation

Neuro-2a cells were collected by trypsinization and resuspended in solution A supplemented with 20 μg/ml leupeptin, 2 μg/ml antipain, and 1 μg/ml pepstatin. For cell disruption, the cell suspension was placed in a 45 ml cell disruption vessel (Parr Instrument Co., Moline, IL) and pressurized with 650 PSI N_2_ gas for 30 min. Cell lysates were collected and centrifuged at 1,000 x *g* for 5 min at 4 °C to remove nuclei and to receive the postnuclear supernatant. The postnuclear supernatant was transferred into Ultra-Clear™ centrifuge tubes (Beckman Instruments, Palo Alto), overlaid with PBS, and centrifuged at 100,000 *g* for 1 h at 4°C. The LD layer on the top, the pellet comprising the membrane fraction, as well as the intermediate supernatant (cytosolic fraction) were collected and subjected to Western blot analysis.

### Cellular imaging

For LD quantification using high-resolution imaging, Neuro-2a cells were cultivated in 8-well chamber slides (Ibidi, Gräfeling, Germany). LDs were labeled using BODIPY 493/503 (Invitrogen, Carlsbad) for 1 h at 37°C (final concentration: 1 μg/ml). Nuclei were labeled using DAPI (Invitrogen, Carlsbad) for 1 h at 37 °C (final concentration: 1 μg/ml). Cell boundaries were labeled using CellMask Deep Red (Invitrogen, Carlsbad). Microscopy was performed on a Leica SP8 confocal microscope (Leica Microsystems Inc., Germany) with spectral detection and using a 63x, HC PL APO CS 63/1.2 NA water immersion objective. DAPI was excited at 405 nm and emission was detected between 410 and 450 nm. BODIPY 493/503 was excited at 488 nm and emission was detected between 500 and 535 nm. CellMask Deep Red was excited at 633 nm and emission was detected between 645 and 750 nm.

Optical sections were acquired using 0.2 μm steps. 3D data were preprocessed using Gaussian filtering (sigma 0.5) and background subtraction (rolling ball radius 15 pixels). LD structures were extracted using the Otsu method in each optical section. Nuclei were manually counted in MIPs. Cells at the border of the images were identified using the CellMask Deep red signal. LDs of such areas were excluded from the analysis. The total LD volume per nuclei was computed. Image processing was performed using the open-source software Fiji (43) and the 3DSuite plug-in. For imaging of ATGL-ECFP, DDHD2-ECFP, and endogenous DDHD2, Neuro-2a were seeded in 8-well chamber slides and incubated for 16 h in the presence of 100 μmol/l oleic acid (complexed to BSA) to induce LD formation. ECFP fusion proteins were detected in living cells. For the detection of endogenous DDHD2 and the Golgi marker protein GM130, cells were fixed for 10 min in PBS containing 4% formaldehyde and permeabilized for 10 min in PBS containing 0.1% Triton X-100. Cells were washed 3 times for 10 min with PBS containing 0.1% Tween-20 (PBT) and blocked with PBT containing 5% goat serum in for 1 h. Cells were then incubated with primary antibodies in PBT containing 5% goat serum for 2 h. Thereafter, cells were incubated with the secondary antibodies in PBT containing 5% goat serum for 1 h. Antibody incubation was followed by three washing steps with PBT for 10 min. The following antibodies were used: mouse anti-GM130 (#610822, BD Biosciences, San Jose), rabbit anti-DDHD2 (#25203-1-AP, Proteintech, Manchester, UK), Rhodamine Red™-linked anti-mouse IgG (#115-295-044, Jackson ImmunoResearch, West Grove), and Dylight® 488-linked anti-rabbit IgG (#PI35552, Thermo Fisher Scientific, Waltham). For the detection of LDs, cells were incubated with HCS LipidTOX™ Deep Red (1:1,000 diluted, Thermo Fisher Scientific) for 20 min. Microscopy was performed on a Leica SP8 confocal microscope (Leica Microsystems Inc., Germany) with spectral detection and using a 63x, HC PL APO CS 63/1.2 NA water immersion objective. The antibody linked to the fluorophore DyLight® 488 was excited at 488 nm and emission was detected between 500 and 540 nm. Rhodamine Red X was excited at 561 nm and emission was detected between 580 and 620 nm. HCS LipidTOX™ Deep Red was excited at 633 nm and emission was detected between 650 and 700 nm.

### Mass spectrometry analyses

For the extraction of DAG from silica TLC plates, 1 μl of internal standard (IST, 8 μmol DAG 17:0, Larodan, Malmö, Sweden) was spotted directly onto the DAG band before it was carefully scrapped off. The silica powder was transferred to fresh 2 ml tubes and extracted with 500 μl methanol by constant shaking at RT for 15 min and subsequent centrifugation at 14,000 rpm for 5 min at RT. Four hundred microliters of the supernatant were transferred to a fresh tube. The silica powder was extracted two more times with 400 μl methyl-*tert*-butyl ether (MTBE)/methanol (3/1, v/v). The supernatants were pooled and evaporated under a stream of nitrogen. The samples were resolved in 500 μl MTBE/methanol (3/1, v/v) and diluted in 2-propanol/methanol/dH_2_O (7/2.5/1, v/v/v) for LC-MS analysis. Cell pellets were extracted according to Matyash *et al.* (44) in 700 μl MTBE/methanol (3/1, v/v) containing 500 pmol butylated hydroxytoluene, 1% acetic acid, and IST (300 pmol PS 34:0, 100 pmol PE 34:0, Larodan; 25 pmol PC 28:0, 25 pmol TAG 45:0, 25 pmol TAG 51:0, Avanti Polar Lipids, Alabaster, ALA). Total lipid extraction was performed under constant shaking for 30 min at room temperature. After addition of 140 μl dH_2_O and further incubation for 10 min at room temperature, the samples were centrifuged at 14,000 rpm for 5 min at RT to ensure proper phase separation. 500 µl of the upper, organic phase were collected and dried under a stream of nitrogen. Lipids were resolved in 500 μl MTBE/methanol (3/1, v/v) and diluted in 2-propanol/methanol/dH2O (7/2.5/1, v/v/v) for LC-MS analysis. The extracted cell proteins were dried and solubilized in NaOH/SDS (0.3 N/0.1%) at 65°C for 4 h and the protein content was determined using the Pierce™ BCA Protein Assay Kit (Thermo Fisher Scientific) and BSA as standard. Chromatographic separation was performed using an Agilent 1290 Infinity II UHPLC (Agilent Technologies, Santa Clara, CA) equipped with a Zorbax Eclipse Plus-C18 rapid resolution high-definition column (2.1 × 50 mm, 1.8 μm; Agilent Technologies), running a 10-min linear gradient from 60% solvent A (H2O; 10 mM ammonium acetate, 0.1% formic acid, 8 μM phosphoric acid) to 100% solvent B (2-propanol; 10 mM ammonium acetate, 0.1% formic acid, 8 μM phosphoric acid). The flow rate was set to 0.5 ml/min and the column compartment was kept at 50°C. An Agilent 6470 triple-quadrupole mass spectrometer with an Agilent Jet Stream ESI (Agilent Technologies) was used for detection. The system was controlled by Agilent MassHunter Acquisition software version 10.1. Data processing was performed with Agilent MassHunter quantitative analysis software version 10.1 and Agilent MassHunter qualitative analysis software version 10.0. Data were normalized to protein and IST and are expressed as arbitrary unit (AU)/IST ratios.

## Statistics

Data were analyzed using GraphPad Prism 9 (GraphPad Software, Sand Diego). Group differences were analyzed by Student’s unpaired *t*-tests and one-way ANOVAs using the Bonferroni correction for multiple comparisons. Differences were considered statistically significant at *P*<0.05. Lipidomic data were analyzed by multiple *t*-tests using the Benjamini-Hochberg method to control for a false discovery rate of 0.05.

## Results

### DDHD2 is a potent acylglycerol hydrolase with a substrate spectrum distinct from other cytosolic lipases

**Fig. 1:**
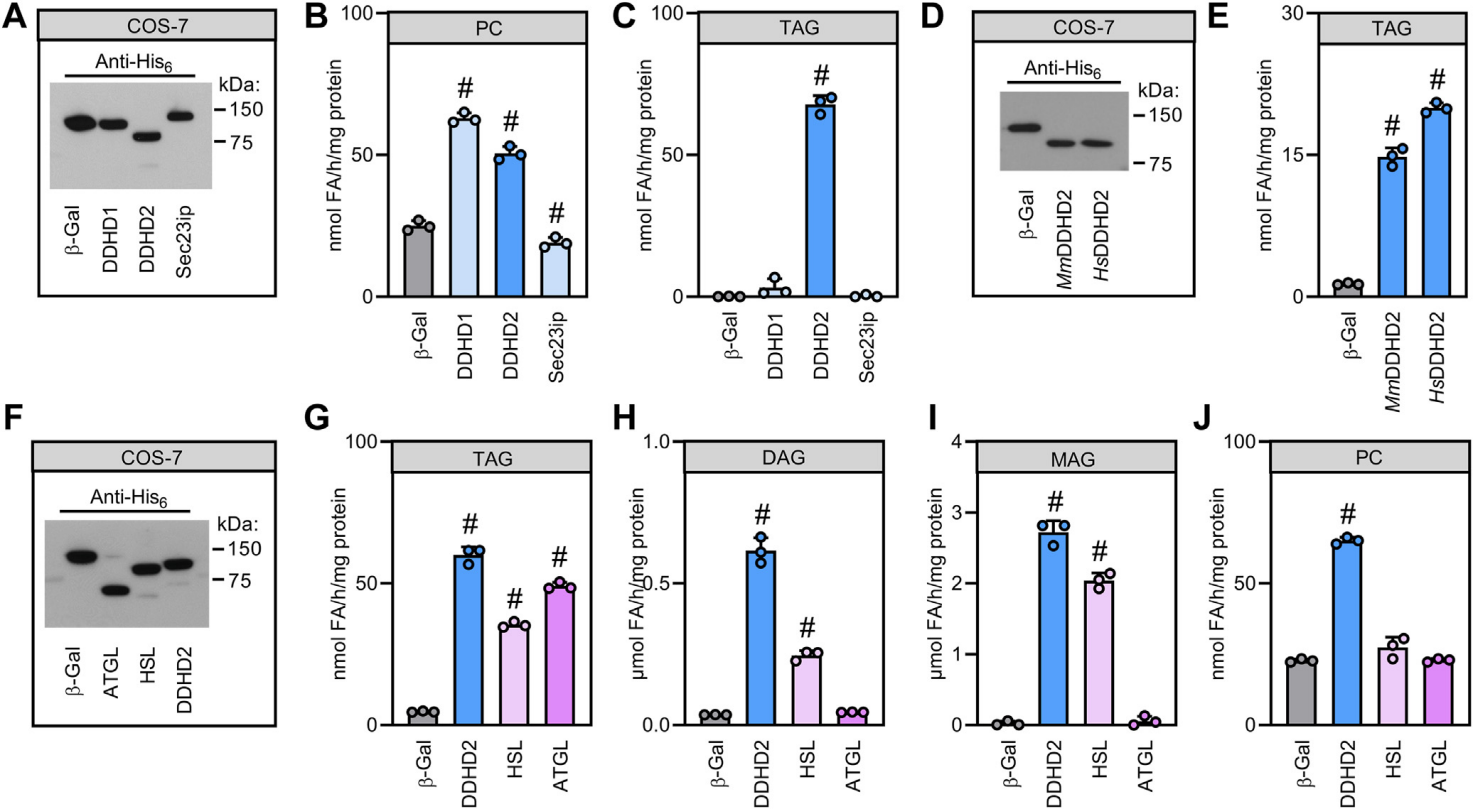
Enzymatic characterization of DDHD2. A, D, and F: Western blotting analysis of COS-7 cell extracts expressing His_6_-tagged recombinant proteins. Equal amounts of cellular protein were resolved by electrophoresis and proteins were detected using an Anti-His_6_ antibody. B, C, E, G, H, I, and J: Lipid hydrolase activities of COS-7 cell extracts expressing His_6_-tagged recombinant proteins. Cell extracts were incubated with lipid substrates as indicated and the release of fatty acids was quantified. Statistical significance was assessed by ANOVA (n = 3; #, *P* < 0.05). ATGL, adipose triglyceride lipase; DAG, diacylglycerol; DDHD1, DDHD domain-containing 1; DDHD2, DDHD domain-containing 2; HSL, hormone-sensitive lipase; MAG, monoacylglycerol; PC, phosphatidylcholine; Sec23ip, Sec23 interacting protein; TAG, triacylglycerol.

To assess lipid hydrolase activities of murine DDHD2 and its paralogs, we expressed His_6_-tagged recombinant DDHD1, DDHD2, and Sec23ip in COS-7 cells and incubated the cellular extracts with emulsified lipid substrates. Western blotting analyses using an antibody directed against the His_6_-tag revealed similar expression levels of DDHD proteins and the negative control β-Galactosidase (β-Gal, Fig. 1A). Consistent with previous reports (26), both DDHD1 and DDHD2 but not Sec23ip increased cellular PC hydrolysis rates by 2.5- and 2-fold, respectively, as compared to controls (Fig. 1B). In addition, DDHD2 exhibited robust TAG hydrolase activity, whereas DDHD1 and Sec23ip did not hydrolyze TAGs (Fig. 1C). Thus, TAG hydrolase activity is specific to DDHD2 and not a general feature of this protein family. We next expressed human and murine DDHD2 in the same cellular setting and found that both enzymes increased TAG hydrolysis to a similar extent as compared to controls indicating that TAG hydrolase activity of DDHD2 has been conserved between mammals (Fig. 1D, E). To compare lipid hydrolase activities of DDHD2 to other cytosolic lipases, we expressed murine DDHD2 in parallel with murine ATGL and murine HSL (Fig. 1F). DDHD2, HSL, and ATGL displayed 12.8-, 7.5-, and 10.4-fold higher TAG hydrolase activities than β-Gal expressing controls (Fig. 1G). Unlike ATGL, expression of DDHD2 and HSL substantially increased hydrolase activities towards DAG (16.8- and 6.7-fold, respectively) and MAG (115- and 86-fold, respectively) when compared to β-Gal-expressing control cell extracts (Fig. 1H, I). Significant PC hydrolase activity was only detected for DDHD2 but not HSL or ATGL (Fig. 1J). Taken together, these data indicate that DDHD2—in addition to its phospholipase activity—has potent acylglycerol hydrolase activities comparable to other cytosolic lipases such as ATGL and HSL.

### DDHD2 determines acylglycerol hydrolase activities in Neuro-2a cells

**Fig. 2:**
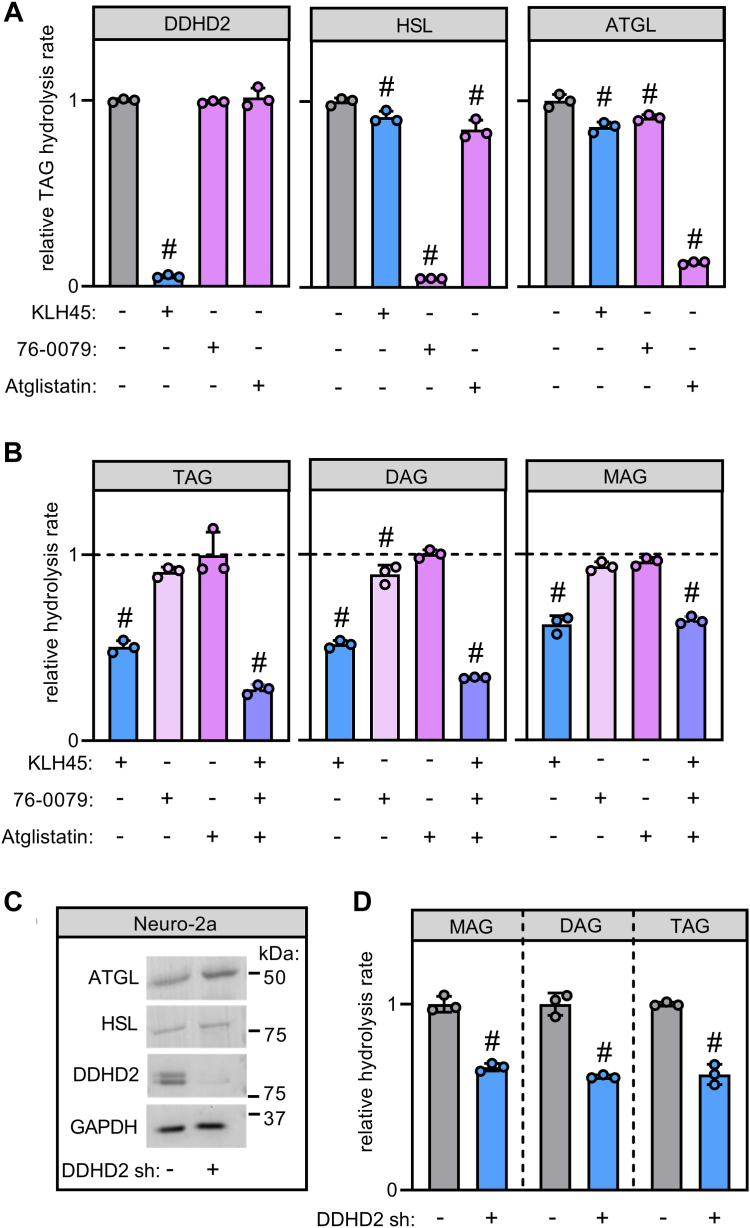
Contribution of cytosolic lipases to acylglycerol hydrolase activities of Neuro-2a cells. A: Inhibition of recombinant lipase activities by small molecules. COS-7 cell extracts expressing His_6_-tagged recombinant enzymes were incubated with TAG substrate in the absence or presence of Atglistatin (40 μM), 76-0079 (1 μM), or KLH45 (100 nM), and the release of fatty acids was quantified. B: Inhibition of endogenous lipase activities in Neuro-2a cells. Cellular extracts were incubated with lipid substrates and small molecule inhibitors as indicated, and the release of fatty acids was quantified. C: Western blotting analysis of lipase expression in Neuro-2a cells. Cells were transduced with lentivirus-expressing scrambled shRNA or DDHD2 shRNA, and protein expression was analyzed using specific antibodies. GAPDH was detected as a loading control. D: Lipid hydrolase activities of Neuro-2a cells after stable silencing of DDHD2 expression. Cellular extracts were incubated with lipid substrates as indicated and the release of fatty acids was quantified. Statistical significance was assessed by ANOVA (n = 3; #, *P* < 0.05). ATGL, adipose triglyceride lipase; DAG, diacylglycerol; DDHD2, DDHD domain-containing 2; GAPDH, glyceraldehyde-3-phosphate dehydrogenase; HSL, hormone-sensitive lipase; MAG, monoacylglycerol; TAG, triacylglycerol.

To understand how DDHD2 interacts with other cytosolic lipases in the regulation of cellular TAG metabolism, we inhibited lipase activity in Neuro-2a neuroblastoma cells using small molecule inhibitors and shRNA-mediated gene silencing. We first assessed the specificity and cross-reactivity of the small molecules KLH45, Atglistatin, and 76-0079, which have been previously used to inhibit DDHD2, ATGL, and HSL, respectively. At a concentration of 100 nM, KLH45 inhibited the TAG hydrolase activity of DDHD2 by 95% but had only marginal effects on the enzyme activities of HSL (8% inhibition) or ATGL (14% inhibition, Fig. 2A). Conversely, 76-0079 and Atglistatin did not affect DDHD2 activity but inhibited activities of HSL and ATGL by 95% and 87%, respectively (Fig. 2A). Next, we used lipase inhibitors to investigate the contribution of each enzyme to Neuro-2a cellular acylglycerol hydrolase activities. Pharmacological inhibition of DDHD2 reduced Neuro-2a cellular hydrolase activities towards TAG, DAG, and MAG by 49%, 48%, and 38%, respectively, as compared to vehicle-treated controls (Fig. 2B). Inhibition of HSL moderately decreased DAG hydrolase activities by 11% with minor effects on MAG and TAG hydrolase activities while inhibition of ATGL did not affect acylglycerol hydrolase activities (Fig. 2B). Combined inhibition of DDHD2, ATGL, and HSL blocked 72% of TAG hydrolase activity and 66% of DAG hydrolase activity (Fig. 2B). These findings indicate that cooperation between cytosolic lipases determines neutral acylglycerol hydrolase activities in Neuro-2a cells with a dominant contribution of DDHD2. To further confirm this observation, we silenced DDHD2 expression by shRNA and measured acylglycerol hydrolase activities. Immunoblotting analysis revealed that stable expression of DDHD2 shRNA strongly reduced DDHD2 but not ATGL or HSL immunoreactivity in Neuro-2a cells (Fig. 2C). Consistently, shRNA-mediated depletion of DDHD2 reduced hydrolase activities towards TAG, DAG, and MAG by 34%–39% as compared to cells treated with control shRNA (Fig. 2D).

### Loss of DDHD2 activity renders Neuro-2a cellular TAG homeostasis functional

**Fig. 3:**
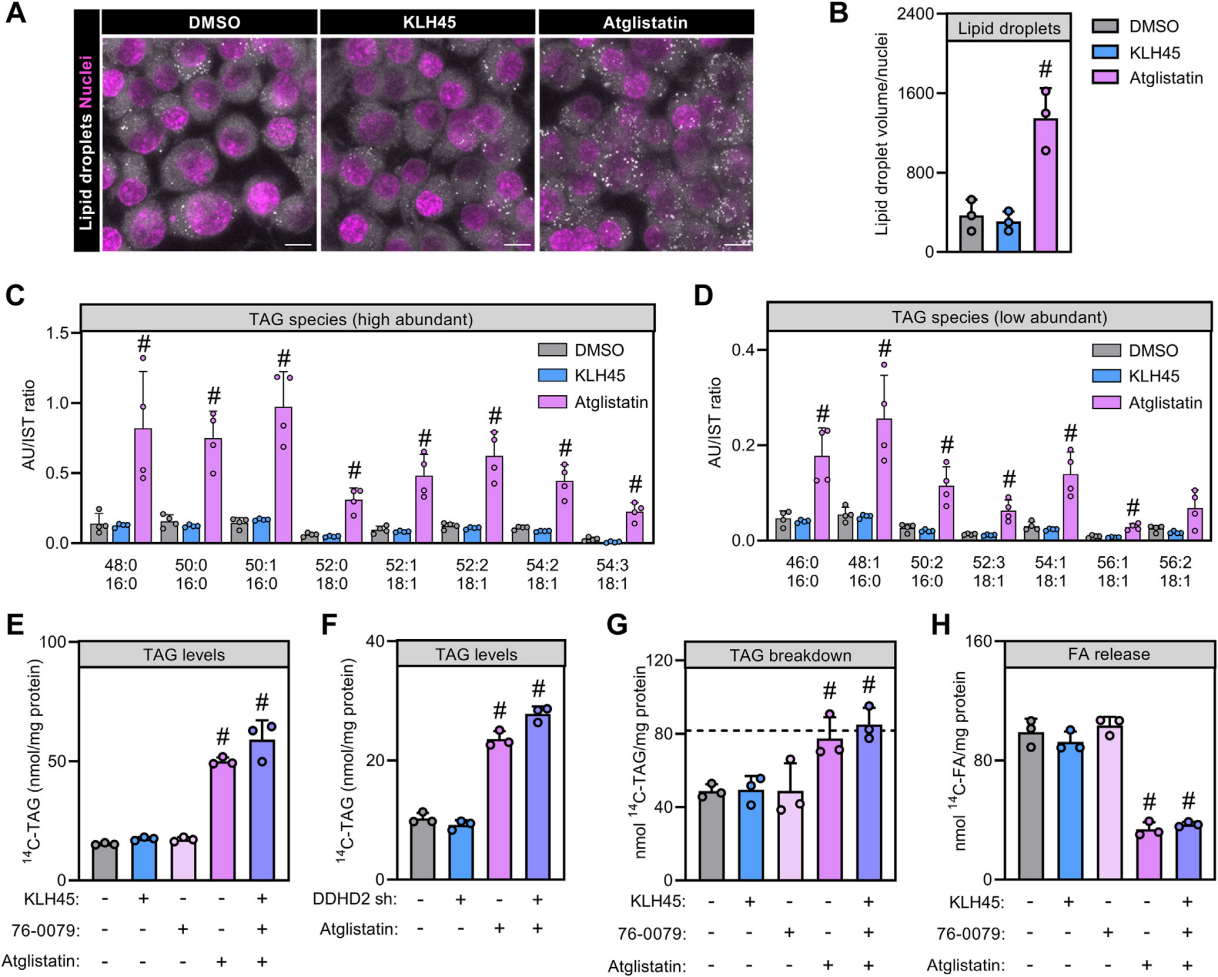
Contribution of cytosolic lipases to LD and TAG homeostasis in Neuro-2a cells. A and B: LD size and abundance in Neuro-2a cells. Cells were incubated for 24 h in the presence and absence of inhibitors and LDs were imaged by confocal fluorescence microscopy. The *left* panel shows a representative Z-stack. Scale bars represent 10 μm. Each datapoint represents a mean of 17–25 cells. C and D: TAG species levels in Neuro-2a cells. Cells were treated with small molecule inhibitors and TAG species were assessed by targeted MS. E and F: TAG levels in Neuro-2a cells incubated with exogenous FAs. Cells were incubated with 400 μM ^14^C-oleic acid in the absence or presence of lipase inhibitors and radioactivity in TAG was determined. G: TAG catabolism and (H) FA release in Neuro-2a cells. Cellular lipids were labeled with ^14^C-oleic acid. Cells were incubated in unlabeled medium in the absence or presence of lipase inhibitors for 4 h, and radioactivity in TAG and secreted FA was determined. The dotted line indicates TAG levels before medium switch. Statistical significance was assessed by ANOVA (n = 3–4; #, *P* < 0.05). TAG, triacylglycerol.

To further understand how lipases determine Neuro-2a acylglycerol homeostasis, we incubated cells with lipase inhibitors and assessed cellular LDs and TAG levels. Pharmacological inhibition of DDHD2 was not associated with an apparent increase in LD abundance or total volume as compared to vehicle-treated controls (Fig. 3A, B). Conversely, inhibition of ATGL increased cellular LD number and caused a 3.7-fold higher total LD volume than vehicle-treated control cells (Fig. 3A, B). Likewise, total TAG levels as well as TAG molecular composition did not change in response to DDHD2 inhibition, while ATGL inhibition broadly increased TAG species with C16:0, C18:0, and C18:1 acyl chains by 2.7- to 7.8-fold as compared to vehicle-treated cells (Fig. 3C, D). This suggests that ATGL rather than DDHD2 limits TAG breakdown in Neuro-2a cells despite the high contribution of DDHD2 to cellular TAG hydrolase activity in vitro. To understand possible cooperation between cytosolic lipases in TAG homeostasis, we incubated cells with specific inhibitors or combinations thereof and followed TAG levels and turnover after lipid labeling with ^14^C-oleic acid. Neuro-2a cells treated with vehicle, HSL inhibitor, or DDHD2 inhibitor exhibited similar TAG levels after incubation with ^14^C-oleic acid. In contrast, inhibition of ATGL increased radiolabeled TAG 3.3-fold as compared to vehicle-treated controls (Fig. 3E). The combined inhibition of all three lipases only slightly increased radiolabeled TAG as compared to ATGL-specific inhibition (3.8- vs. 3.3-fold compared to controls) suggesting a dominant role of ATGL in cellular TAG homeostasis (Fig. 3E). To corroborate these findings, we repeated the experiment after silencing DDHD2 expression with shRNA. Consistently, depletion of DDHD2 failed to increase cellular radiolabeled TAG levels in the basal state or in combination with ATGL inhibition (Fig. 3F). Finally, we directly assessed lipolysis by measuring the breakdown of cellular TAG and the release of FA into the medium using delipidated BSA as FA scavenger. Inhibition of ATGL, but not of DDHD2 or HSL, attenuated TAG consumption and FA release as compared to vehicle-treated control cells (Fig. 3G, H). Combined inhibition of all lipases did not further decrease TAG lipolysis rates as compared to inhibition of ATGL alone (Fig. 3G, H). Taken together, these data indicate a dominant role of ATGL in TAG and LD catabolism of Neuro-2a cells.

### DDHD2 hydrolyzes LD-derived DAG but does not affect phospholipid composition in Neuro-2a cells

**Fig. 4:**
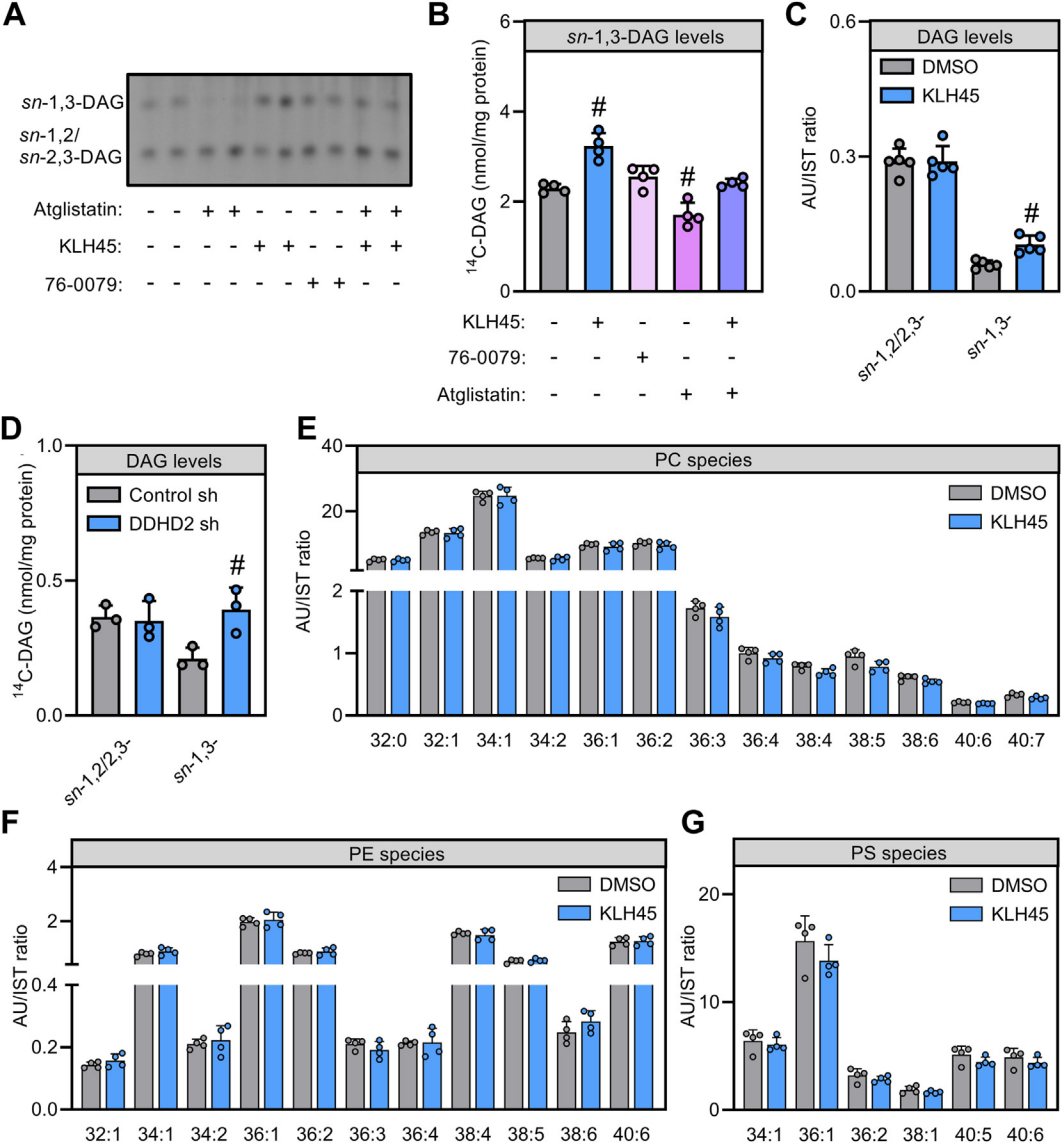
Impact of cytosolic lipases on DAG pools and other lipids in Neuro-2a cells. A, B, and D: Levels of DAG regiomers in Neuro-2a cells. Cellular lipids were labeled with ^14^C-oleic acid in the presence and absence of inhibitors and radioactivity in DAG regiomers was visualized by autoradiography and quantified thereafter. Cells in (D) were transduced with lentivirus-expressing scrambled or DDHD2 shRNA. (C) Levels of DAG 36:2 regiomers in Neuro-2a cells. Cells were incubated in the absence or presence of inhibitors, and DAG 36:2 regiomers were quantified by targeted MS. E, F, and G: Phospholipid levels in Neuro-2a cells. Cells were incubated in the absence or presence of lipase inhibitors, and cellular PC, PE and PS species were quantified by targeted MS analysis. Statistical significance was assessed by ANOVA (n = 3–5; #, *P* < 0.05). DDHD2, DDHD domain-containing 2; DAG, diacylglycerol; PC, phosphatidylcholine; PE, phosphatidylethanolamine; PS, phosphatidylserine.

Since DDHD2 broadly affects cellular neutral lipid hydrolase activities, we extended our tracer experiments to test if DDHD2 affects intracellular DAG metabolism. DAG exists in three regiomers: *sn*-1,2 DAG, *sn*-2,3 DAG, and *sn*-1,3 DAG. *sn*-1,2 DAG is an intermediate of glycerolipid synthesis pathways and glycerophospholipid breakdown by phospholipase C and phosphatidate phosphatase. Conversely, intracellular *sn*-1,3 DAG (and *sn*-2,3-DAG) is predominantly formed by ATGL-mediated TAG hydrolysis (45). To separate *sn*-1,3-DAG from *sn*-1,2/2,3-DAG, we labeled cellular lipids by ^14^C-oleic acid and assessed the effect of lipase inhibition on DAG regiomers by thin layer chromatography (Fig. 4A, B). Pharmacological inhibition of DDHD2 increased radiolabeled *sn*-1,3-DAG 1.4-fold as compared to vehicle-treated controls. While inhibition of HSL did not affect *sn*-1,3-DAG levels, inhibition of ATGL specifically decreased *sn*-1,3-DAG levels consistent with the notion that *sn*-1,3-DAG derives from ATGL-mediated TAG breakdown (Fig. 4A, B). Notably, ATGL inhibition also prevented the increase in *sn*-1,3-DAG by KLH45 indicating that ATGL supplies *sn*-1,3-DAG to DDHD2 (Fig. 4A, B). While *sn*-1,3-DAG levels responded to lipase inhibition, *sn*-1,2/2,3-DAG was largely unaffected by altering lipase activity (Fig. 4A). Targeted MS analysis of *sn*-1,3-DAG and *sn*-1,2/2,3-DAG fractions confirmed that DDHD2 inhibition selectively increased *sn*-1,3-DAG levels 1.8-fold in Neuro-2a cells without apparent effects on *sn*-1,2/2,3-DAG (Fig. 4C). To exclude that off-target effects of KLH45 accounted for the observed alteration in DAG metabolism, we analyzed DAG isomers in Neuro-2a after shRNA-mediated silencing of DDHD2 expression. Consistently, depletion of DDHD2 was associated with 1.9-fold increased *sn*-1,3-DAG but similar *sn*-1,2/2,3-DAG levels as compared to control cells (Fig. 4D). Given that DDHD2 is a potent phospholipase (26, 28), we asked if DDHD2 specifically controls DAG or has broader effects on the cellular lipidome. Therefore, we assessed the levels of several major glycerophospholipid species in the absence or presence of KLH45. However, overall cellular levels and composition of PC, phosphatidylethanolamine (PE), or phosphatidylserine (PS) did not significantly change in response to DDHD2 inhibition (Fig. 4E–G). Taken together, these data indicate that in Neuro-2a cells, DDHD2 acts downstream of ATGL as a specific regulator of *sn*-1,3-DAG.

### ATGL and DDHD2 cooperate in primary neuronal TAG catabolism

**Fig. 5:**
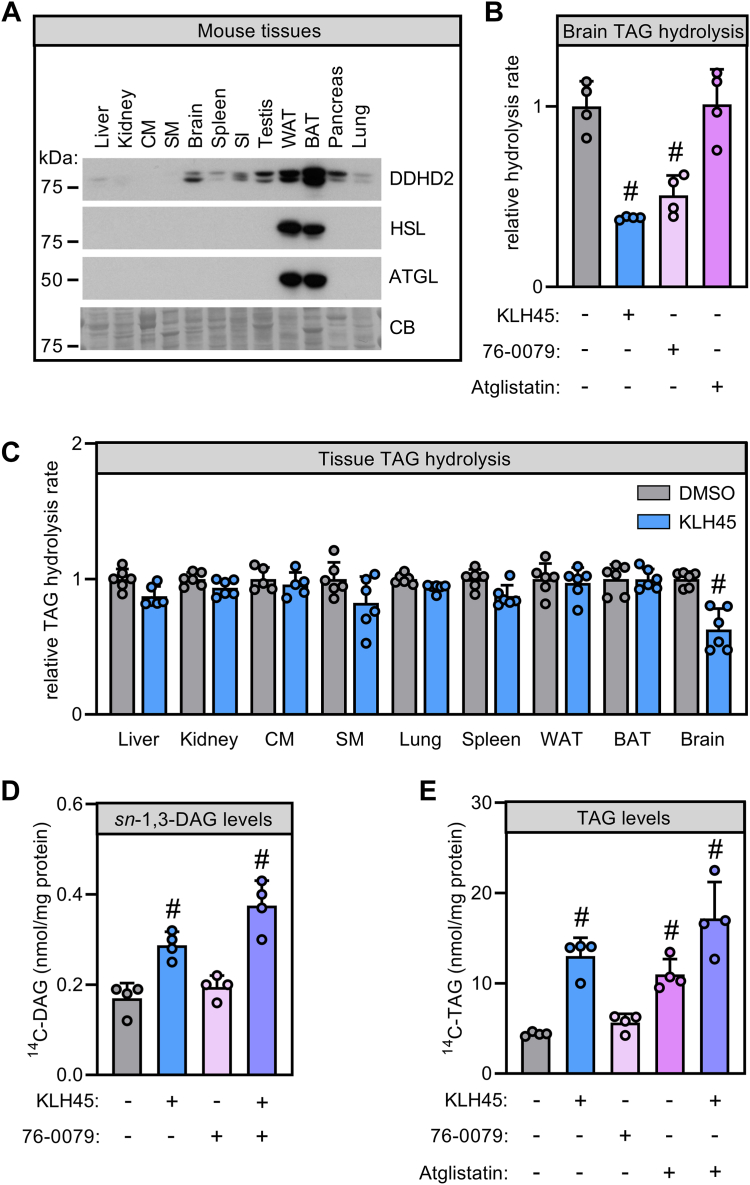
Impact of cytosolic lipases on TAG hydrolysis of murine brain and primary cortical neurons. A: Western blotting analysis of cytosolic lipase expression in murine tissues. Tissue homogenates of male C57BL/6J were separated by gel electrophoresis, and protein expression of DDHD2, HSL, and ATGL was detected by immunoblotting. B: Inhibition of endogenous lipase activities in murine brain homogenates. Brain extracts were incubated with TAG substrates and small molecule inhibitors as indicated, and the release of fatty acids was quantified. C: Inhibition of DDHD2 activity in murine tissue homogenates. Tissue extracts were incubated with TAG substrates and KLH45 or DMSO as indicated, and the release of fatty acids was quantified. D: Levels of sn-1,3-DAG and (E) TAG in primary cortical neurons after lipase inhibition. Primary cortical neurons were incubated with ^14^C-oleic acid and lipase inhibitors as indicated, and radioactivity in *sn*-1,3-DAG and TAG, respectively, was quantified. Data are expressed as means +SD. Statistical significance was assessed by (C) multiple t-tests or (B, D, E) ANOVA (n = 4–6; #, *P* < 0.05). TAG, triacylglycerol; ATGL, adipose triglyceride lipase; BAT, brown adipose tissue; CM, cardiac muscle; DDHD2, DDHD domain-containing 2; DAG, diacylglycerol; HSL, hormone sensitive lipase; SI, small intestine; SM, skeletal muscle; TAG, triacylglycerol; WAT, white adipose tissue.

To extend our findings in the neuroblastoma cell model, we next assessed the effect of lipase inhibition on brain TAG hydrolase activities and TAG metabolism of primary cortical neurons. Immunoblotting analysis revealed that DDHD2 is broadly expressed in brain and peripheral tissues (Fig. 5A). While expression of ATGL and HSL has also been previously detected in brain, both proteins are strongly enriched in adipose tissue as compared to DDHD2 (Fig. 5A) (37, 46). Inhibition of DDHD2 decreased brain TAG hydrolase activity by 62% as compared to vehicle-treated controls (Fig. 5B). Likewise, HSL inhibition reduced brain TAG hydrolase activity by 49% while inhibition of ATGL did not affect brain TAG hydrolase rates, indicating a dominant contribution of DDHD2 (Fig. 5B). Intriguingly, the impact of DDHD2 on TAG hydrolysis was essentially restricted to brain as inhibition of DDHD2 activity only marginally decreased TAG hydrolase activities of peripheral tissues (Fig. 5C). We next isolated primary cortical neurons from mouse neonates and incubated them with ^14^C-oleic acid in the presence of lipase inhibitors to assess their impact on neuronal acylglycerol metabolism. Similar to Neuro-2a cells, inhibition of DDHD2 elevated *sn*-1,3-DAG in primary neurons 1.7-fold as compared to vehicle-treated controls consistent with a function as DAG lipase (Fig. 5D). Conversely, inhibition of HSL—a major adipose DAG lipase—did not affect neuronal *sn*-1,3-DAG (Fig. 5D). Moreover, DDHD2 inhibition also increased neuronal TAG 3-fold as compared to controls (Fig. 5E). Similarly, ATGL but not HSL inhibition elevated cellular TAG 2.5-fold. Combined inhibition of ATGL and DDHD2 further increased neuronal TAG, resulting in a 3.9-fold increase in TAG level as compared to controls (Fig. 5E). Taken together, these results indicate that DDHD2 acts as a dual DAG/TAG lipase in neurons and cooperates with ATGL to regulate neuronal TAG hydrolysis.

### DDHD2 localizes to the cytosol and Golgi compartment

**Fig. 6:**
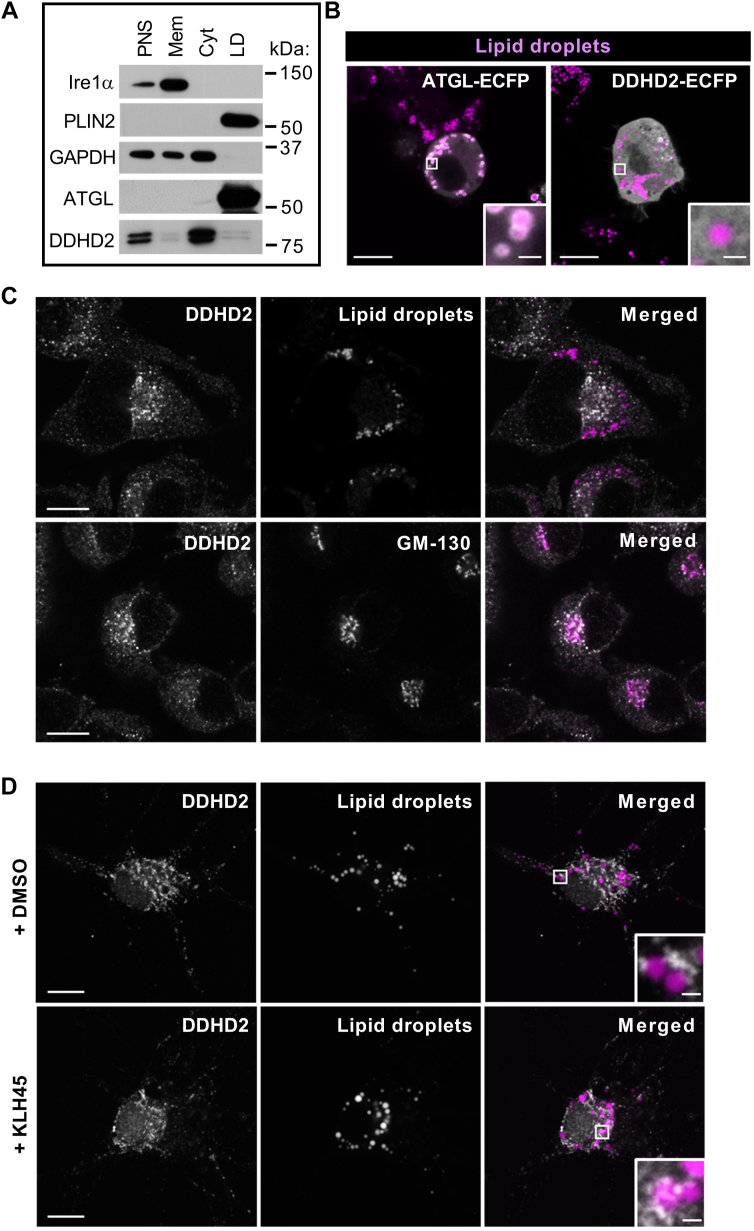
Subcellular localization of cytosolic lipases in Neuro-2a cells and primary cortical neurons. Cells were incubated with oleic acid to induce lipid droplet (LD) formation. A: Neuro-2a perinuclear supernatants (PNS) were separated into cytosol (Cyt), membrane (Mem), and LD fractions by ultracentrifugation, and protein distribution was assessed by Western blotting analysis. B: subcellular localization of recombinant lipases in living Neuro-2a cells. Cells transiently expressing ATGL-ECFP or DDHD2-ECFP were incubated with oleic acid to induce LD formation and imaged by confocal fluorescence microscopy. LDs were detected using LipidTOX. Scale bars full size: 10 μm; scale bars insets: 1 μm. C, D: subcellular localization of endogenous DDHD2 in (C) Neuro-2a cells and (D) primary mouse neurons. Cells were incubated in the presence of oleic acid and in the absence or presence of KLH45 as indicated. Cells were fixed, and DDHD2 was detected by immunocytochemistry. LDs and *cis*-Golgi were detected using LipidTOX and anti-GM130 antibody, respectively. Scale bars full size: 10 μm; scale bars insets: 1 μm. ATGL, adipose triglyceride lipase; DDHD2, DDHD domain-containing 2; GAPDH, glyceraldehyde-3-phosphate dehydrogenase; Ire1α; inositol-requiring enzyme 1α; PLIN2, perilipin 2.

Lipolysis requires interaction of enzymes with the surface of LDs. To understand how DDHD2 gains access to *sn*-1,3-DAG and TAG substrates, we compared the subcellular localization of ATGL and DDHD2 by biochemical fractionation and imaging approaches. Endogenous ATGL was highly enriched in LD fractions of Neuro-2a cells obtained by ultracentrifugation resembling the LD marker protein perilipin 2 (PLIN2) (Fig. 6A). Conversely, DDHD2 was recovered mostly in the cytosolic fraction with little immunoreactivity in the LD fraction resembling the cytosolic marker protein GAPDH. Consistent with this, recombinant ECFP-tagged ATGL colocalized with LDs in living Neuro-2a cells as assessed by confocal fluorescence imaging, whereas ECFP-tagged DDHD2 exhibited a cytosolic localization (Fig. 6B). Moreover, endogenous DDHD2 protein was enriched in the perinuclear region of Neuro-2a cells and localized in close proximity to the Golgi protein GM130 but not to LDs (Fig. 6C). In primary cortical neurons, endogenous DDHD2 immunoreactivity decorated vesicular and reticulate structures resembling a pattern previously reported for GM130 (47). LDs were often found in close proximity to DDHD2-positive structures in particular after pharmacological DDHD2 inhibition (Fig. 6D). Yet, DDHD2 did not decorate the LD surface. We conclude that DDHD2—unlike ATGL—does not permanently associate with LDs but accesses TAG and *sn*-1,3-DAG substrates via organelle contact sites or is transiently recruited form the cytosol by unknown mechanisms.

## Discussion

The “classical” lipolysome of adipocytes consists of ATGL, HSL, MGL, and numerous regulatory proteins whose interplay controls the sequential hydrolysis of LD-associated TAG. The existence of additional lipolytic enzymes in several nonadipose tissues suggests alternative lipolysomes whose configurations are often incompletely understood (18, 19, 20, 21, 24). The enzyme DDHD2, initially annotated as phospholipase A1, was previously identified as a principal neuronal TAG hydrolase and core component of the neuronal lipolysome (24, 25). Global loss of *Ddhd2* in mice leads to accumulations of TAG and LDs specifically in neurons but not in peripheral tissues such as cardiac muscle or liver (24). Likewise, abnormal neutral lipid accumulations in the brain of patients with loss-of-function mutations in the *DDHD2* gene (33, 48) indicate a primary function of human DDHD2 in neuronal TAG catabolism. Consistent with these observations, our experiments reveal that inhibition of DDHD2 substantially reduces murine brain TAG hydrolase activity and leads to TAG accumulations in primary mouse neurons. An important novel finding of our study is that DDHD2 functionally interacts with ATGL in neuronal TAG hydrolysis. Inhibition of ATGL per se was sufficient to increase TAG levels in primary neurons and combined inhibition of both ATGL and DDHD2 further increased neuronal TAG.

Furthermore, we identified a dominant role of ATGL in intracellular lipolysis of Neuro-2a neuroblastoma cells, in which DDHD2 adopted a more restricted lipolytic function in DAG hydrolysis downstream of ATGL. Even though DDHD2 inhibition in Neuro-2a cell extracts reduced in vitro TAG hydrolase activity by approximately 50%, DDHD2 inhibition or knockdown in living Neuro-2a cells did not influence TAG content or LD abundance. Several explanatory approaches can be found for this discrepancy between DDHD2’s enzymatic behavior in vitro and in living cells. First, artificial TAG particles offered in our assays deviate from natural LDs in terms of biophysical properties, chemical composition, and attached surface proteins. It is thus conceivable that the (phospho-) lipid composition or curvature of Neuro-2a LDs hinders the access of DDHD2 to its TAG substrate. Given the fact that the proteome of different brain regions is highly cell type-specific (25, 49), Neuro-2a cells (but not primary cortical neurons) might lack currently unknown protein interaction partners required for DDHD2-mediated TAG hydrolysis. Second, our microscopic analyses revealed that DDHD2 localized primarily in the perinuclear region of Neuro-2a cells in proximity to the *cis*-Golgi marker GM130 but not with LDs. This restricted localization pattern might prevent DDHD2 from accessing the TAG substrate. Conversely, DDHD2-immunopositive structures in primary cortical neurons were more interspersed with LDs likely facilitating interactions of the enzyme to the LD surface.

Collectively, these findings indicate that DDHD2 and ATGL act in close cooperation in neuronal lipolysis. ATGL expression has been previously detected in the murine brain including certain neurons, but ATGL deficiency in mice or humans has not been associated with widespread neuronal TAG accumulation (46). Moreover, unlike patients with SPG54 due to *DDHD2* mutations, patients with loss-of-function mutations in the *PNPLA2/ATGL* gene are rarely diagnosed with CNS disorders (50). These phenotypic differences in mouse models and patients suggest a dominant role of DDHD2 over ATGL in the degradation of neuronal TAG in vivo. Hence, the contribution of ATGL to neuronal TAG catabolism in vivo might be restricted to specific developmental stages, (patho)physiological conditions, or cell populations. Several additional observations support a role of ATGL in neuronal TAG catabolism: ATGL is expressed in neural stem cells and isolated spinal ganglion neurons, and inhibition of its enzyme activity in those cells increases LD abundance in vitro (51). Moreover, silencing of murine *Atgl* impairs axonal regeneration after optical nerve injury in vivo (52). Finally, the *Caenorhabditis elegans* ATGL homolog Atgl-1 and the *Drosophila* ATGL homolog Brummer both regulate neuronal LDs in vivo (53, 54) indicating an ancestral role of this protein family in neuronal lipolysis.

Our comparative assays as well as previous studies revealed that recombinant DDHD2 exhibits an extremely broad substrate spectrum beyond TAG and hydrolyzes multiple phospholipids and acylglycerols in vitro. Reductions in neuroblastoma DDHD2 expression or activity broadly reduced cellular acylglycerol hydrolysis rates towards MAG, DAG, and TAG. Accordingly, inhibition of DDHD2 in primary neurons increased not only TAG but also *sn*-1,3 DAG, which are intermediates of the lipolytic process and specific products of ATGL activity (45, 55). Consistently, elevated *sn*-1,3 DAG levels were a primary consequence of DDHD2 inhibition in neuroblastoma cells. This suggests that DDHD2 can act as dual TAG/DAG lipase allowing its engagement at different steps of the lipolytic cascade. The relaxed substrate spectrum and functional flexibility of DDHD2 resembles that of HSL in peripheral lipolysis. Like DDHD2, HSL accepts multiple lipid substrates in vitro and adopts different roles in the lipolysome depending on the cellular context and cooperation with other lipolytic enzymes (56, 57). In brown adipocytes, HSL acts downstream of ATGL in DAG hydrolysis, yet also contributes to TAG hydrolysis in parallel with ATGL (6, 56). This configuration resembles the interplay of ATGL and DDHD2 in neuronal lipolysis. Thus, while neuronal and peripheral lipolysomes differ in composition, the biochemical interactions of the enzymes involved are similar. DDHD2 is present in several peripheral tissues (26) and is frequently co-expressed with ATGL, HSL, or other lipolytic enzymes. Ectopic TAG accumulations have not been reported in peripheral tissues of *Ddhd2* knockout mice (24). Nevertheless, given its intrinsic substrate promiscuity, it is conceivable that in certain peripheral tissues, DDHD2 participates in lipolysis by hydrolyzing DAG and MAG.

TAG-rich LDs have rarely been detected in neurons from young, healthy brains but accumulate upon stress, aging, and neurodegeneration (58). Nonetheless, there appear to be substantial turnover rates of neuronal TAG under physiological conditions, as pharmacological DDHD2 inhibition increases murine brain TAG after only a few days (24). The biological role of neuronal lipolysis and how disruption of it leads to neuronal dysfunction is yet unknown. Recent studies in *C. elegans* and a mouse model of optical nerve injury suggest that neuronal lipolysis aids in FA partitioning towards membrane lipid synthesis (52, 54). Such TAG pools may be spatially and temporally highly restricted. When not appropriately controlled LDs may spherically interfere with proper positioning of other neuronal organelles or optimal secretion of axonal cargo. Neurons also have a limited capacity for FA oxidation and are rich in polyunsaturated FAs, which are prone to peroxidation. The stockpiling of FAs in TAG may thus increase the risk of lipotoxic injury to neurons. Several elegant studies demonstrated that under stressed conditions, like redox imbalance, neuronal lipids are actively expelled rather than being stored as TAG (58, 59). The expelled lipids are then transported via ApoE carriers into neighboring astrocytes, where they induce LD formation. Dual lipolytic control over neuronal TAG via DDHD2 and ATGL may reflect a specific requirement of neurons to limit LD abundance and size or to streamline stress-induced intercellular lipid transfer. Our studies reveal that both enzymes target different subcellular structures with privileged access of ATGL to cellular LDs and may thus metabolize distinct TAG pools. While ATGL acts at the LD surface, DDHD2 may be active on TAG pools in bilayers of perinuclear endomembranes or on nascent LDs diverting lipids towards secretion at early steps of LD formation. Nevertheless, the precise role of each lipolytic enzyme in this process and how they contribute to neuronal function under stressed condition awaits to be further characterized. In summary, our study indicates that DDHD2 participates in neuronal lipolysis as dual TAG/DAG lipase in close cooperation with ATGL and highlights a previously unrecognized complexity of the neuronal lipolysome.

## Data Availability

All data generated or analyzed during this study are included in the manuscript and supporting files.

## Conflict of interest

The authors declare that the research was conducted in the absence of any commercial or financial relationships that could be construed as a potential conflict of interest.
